# Correlation between various trace elements and ultramicroscopic structure of epiretinal macular membranes and glial cells

**DOI:** 10.1371/journal.pone.0204497

**Published:** 2018-09-28

**Authors:** Mario R. Romano, Gilda Cennamo, Daniela Montorio, Salvatore Del Prete, Mariantonia Ferrara, Giovanni Cennamo

**Affiliations:** 1 Department of Biomedical Sciences, Humanitas University, Pieve Emanuele—Milan, Italy; 2 Department of Public Health, University Federico II, Naples, Italy; 3 Department of Neuroscience, Reproductive and Odontostomatological Science, University Federico II, Naples, Italy; 4 Interdepartment Electron Microscope Centre, University Federico II, Naples, Italy; Massachusetts Eye & Ear Infirmary, Harvard Medical School, UNITED STATES

## Abstract

**Introduction:**

Elements such as zinc, iron, copper, sulphur and phosphorus have been identified in retinal layers and implicated in vital retinal functions. Regarding mineral composition of epiretinal membranes (ERMs), literature is lacking. This study aimed to analyze both mineral composition and anatomical ultrastructure of ERMs to clarify the pathophysiology of this disease.

**Methods:**

Twenty ERMs (10 diabetic ERMs and 10 idiopathic ERMs) from 20 patients were harvested during pars plana vitrectomy. Scanning Electron Microscopy (SEM) was used to investigate the anatomical ultrastructure of the peeled ERMs. Mineral composition was analyzed using energy-dispersive spectrometry (EDS). The most frequent elements were evaluated in relation to appearance of ERMs analyzed at SEM and at OCT images.

**Results:**

Sulphur was the most frequent element found (in 80% of the samples), followed by sodium (50%) and phosphorus (45%). The presence of these elements was not significantly different between diabetic and idiopathic ERMs (P >0.05). Using SEM we found a folded tissue in all ERMs, except in 4 ERMs, where we observed only a smooth tissue. There was a trend of sodium to be more frequent in ERMs with folded layers at SEM examination.

**Conclusions:**

Several elements were identified in ERMs, and sulphur, sodium and phosphorus were the most frequent ones. This finding may help to understand their role in the physiopatology of epiretinal proliferation and in glial activation.

## Introduction

Epiretinal membrane (ERM) is the most common type of fibrocellular proliferation at the vitreoretinal interface and is significantly associated with aging [1–3].

Several previous studies aimed to identify the cell types in ERMs using light and electron microscopy [4–8]. However, during the last decades, morphologic analyses of surgically excised ERM specimens were inadequate because of the phenotypic trans-differentation of proliferating epiretinal cells [9–11]. Showing the presence of glial cells (Muller cells, fibrous astrocytes, microglia), fibroblasts, myofibroblasts, hyalocites, retinal pigment epithelial cells and macrophages, recent immunohistochemical investigations confirmed the involvement of these cells in ERM formation [12–14].

Recently Azzolini et al. [15] observed the appearance of iERMs at scanning electron microscopy (SEM), identifying four types of structures distributed in various layers from ILM to vitreous side of the membranes. In particular, the Authors described: (a) thin layers of woven fibers; (b) folded layers of fibrous material; (c) rigid, thicker and more densely folded layers of collagen fibrils; and (d) necrotic and/or inflammatory material in lacunar structures.

Previous studies investigated the presence of mineral elements in the retinal layers because of their role in various retinal diseases [16]. It has been demonstrated that the altered homeostasis of zinc and iron is implicated in retinal dysfunction and age-related macular degeneration [17–18], as well as copper deficiency in optic neuropathy and altered zinc levels in poor dark adaptation [17].

As far as we know, in literature there is no study regarding mineral composition of ERMs.

Our purpose is to investigate both anatomical ultrastructure and mineral composition of ERMs, in order to improve the understanding of the physiopathology of this disease.

## Materials and methods

In this prospective study we evaluated 20 ERMs of 20 consecutive patients enrolled in the Eye Clinic of the University of Naples “Federico II” from July to October 2016. Before undergoing surgery, all patients signed a written informed consent. The study was approved by the Institutional Review Board of the University of Naples “Federico II” and all investigations adhered to the tenets of the Declaration of Helsinki. We included 10 idiopathic ERMs (iERMs) and 10 ERMs secondary to diabetic retinopathy (dERMs). Exclusion criteria were previous ophthalmic laser and surgical treatment, intravitreal injection, vascular occlusions, inflammatory eye diseases, history of ocular trauma and significant ocular media opacities precluding an adequate fundus and optical coherence tomography (OCT) examination.

All patients underwent best corrected visual acuity (BCVA) test by Snellen eye chart, slit-lamp biomicroscopy, dilated fundus examination, Spectral Domain-OCT by RTVue-100 OCT XR Avanti (Optovue Inc., Fremont, CA, USA; software version 4.0.5.39) and Spectralis OCT (Heidelberg Engineering, Heidelberg, Germany) with multimodal imaging. Based on fundus examination, multicolor and infrared images, we categorized ERMs according Gass’s classification. (REF)

The 20 eyes underwent 25-gauge pars plana vitrectomy and ERM peeling dye-assisted.

Immediately after their removal, ERM specimens were fixed in 3% glutaraldehyde in a 0.065 M (pH 7.4) phosphate buffer for two hours at room temperature. Slides were washed three times in 0.065 M phosphate buffer (for 30 minutes), then placed in 1% OsO4 in 0.065 M (pH7.4) phosphate buffer for 30 minutes. The samples were dehydrated through a graded series of ethanol, and then critical-point-dried in a CO2 liquid Bemar SPC 1500 apparatus (Bomar Co, Tacome, WA, USA). Specimens were mounted on aluminium stubs, placed into molecular coating with graphite and examined using SEM JEOL (JSM 5310).

Mineral composition was analysed using energy-dispersive X-ray spectrometry (EDS) with an EDS detector (Oxford Instruments-INCA). Qualitative EDS analysis was performed using the ‘automatic peak identification’ software. When invoked, automatic peak identification applies a mathematical algorithm to locate and measure the photon energy of the characteristic peaks in the spectrum and then assigns elemental labels from a database of elemental X-ray energy information [19]. The slides and the dyes were also analyzed EDS in order to exclude any interference in mineral evaluation of ERMs.

### Statistical analysis

Statistical analysis was performed using the Statistical Package for Social Sciences (Version 20.0 for Windows; SPSS Inc, Chicago, Ill, USA). The Fisher’s exact test was used to evaluate if the difference in the presence of the most frequent elements was significant between iERMs and dERMs. A p value < 0.05 was considered statistically significant.

## Results

Twenty ERMs surgically removed from 20 eyes of 20 patients (10 females and 10 males) were examined. The mean age was 65.5 ± 9.77 years and the mean preoperative BCVA was 0.66 ±0.18 logMAR. Ten of 20 patients were affected by dERM and 10 by iERM.

All ERMs were classified according to Gass’s criteria [1]: 65% was grade I (6 dERMs, 7 iERMs); 35% grade II (4 dERMs, 3 iERMs).

**Fig 1:**
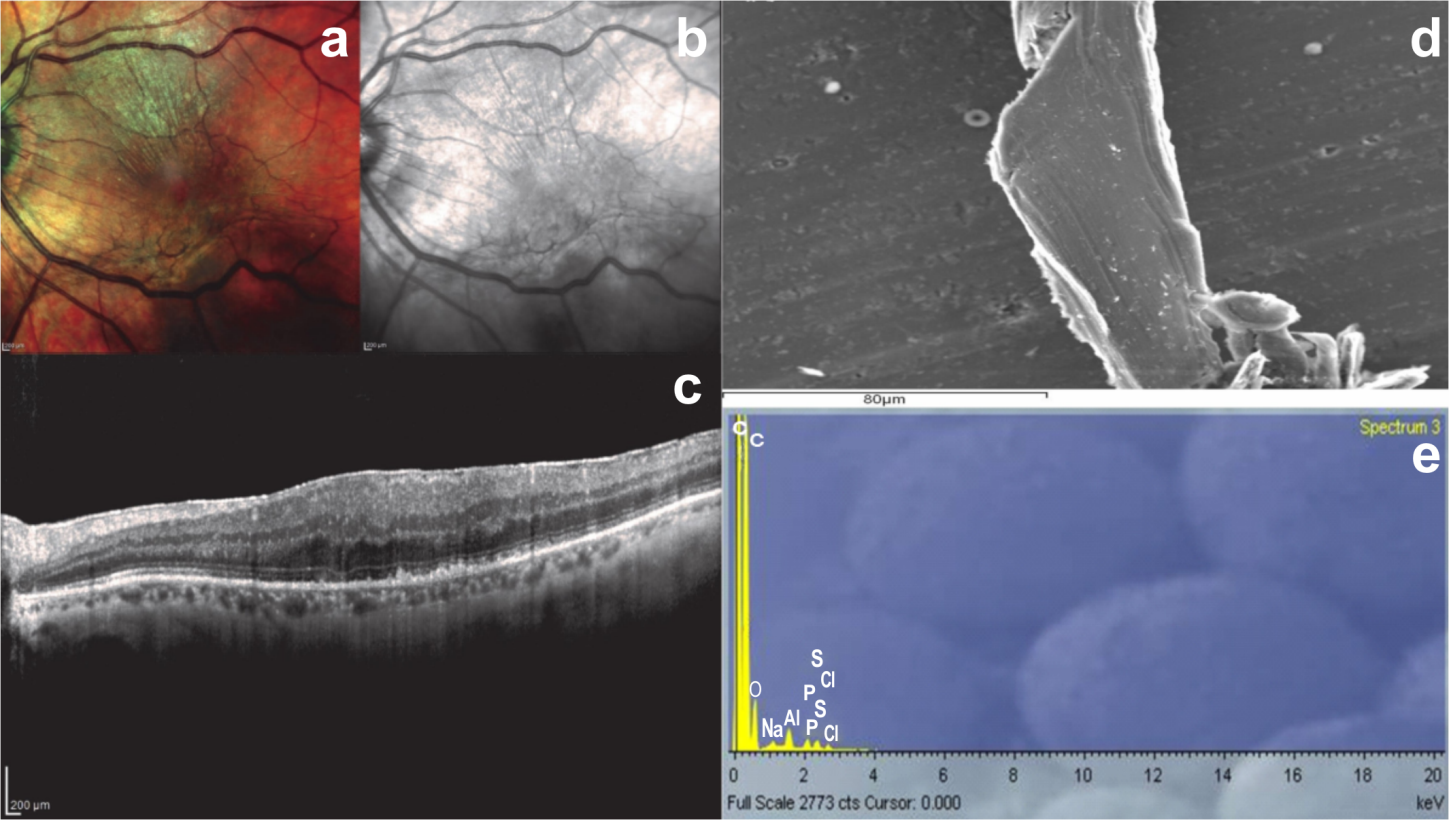
Analysis of a sample ERM with smooth appearance at SEM. a-c) Multicolor image, infrared and structural OCT B-scan showing slight epiretinal proliferation; d) Scanning electron microscope sample revealing smooth tissue; e) SEM/EDS analysis showing the mineral composition of the sample: sulphur, sodium and phosphorus are the most frequent elements.

**Fig 2:**
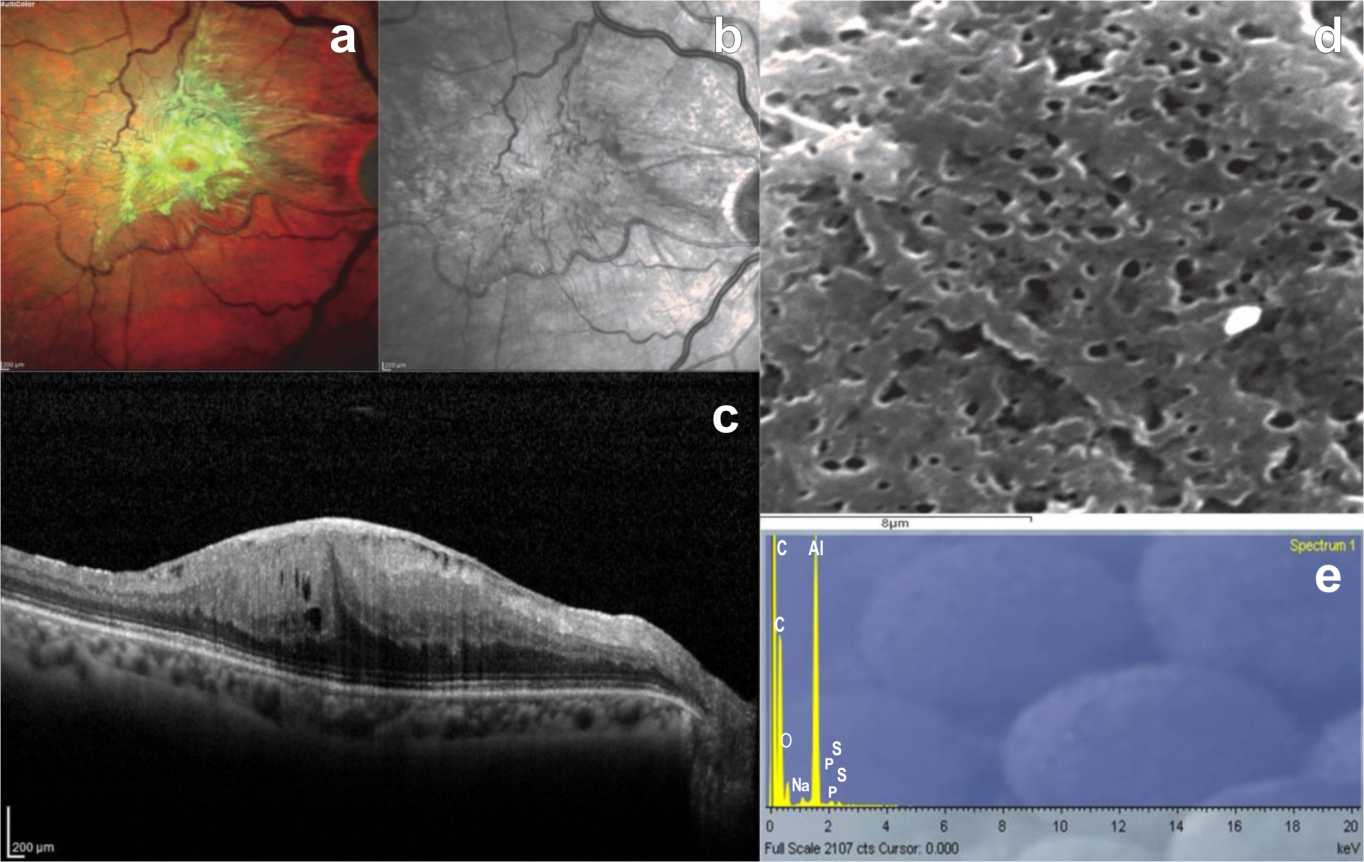
Analysis of a sample ERM with folded appearance at SEM. a-c) Multicolor image, infrared and structural OCT B-scan showing significant epiretinal proliferation; d) Scanning electron microscope sample revealing fibrotic folded tissue; e) SEM/EDS analysis showing the mineral composition of the sample: sulphur, sodium and phosphorus are the most frequent elements.

We observed all ERMs at SEM. According to the structures recently described by Azzolini et al. [15], we found a smooth appearance due to only thin layers of woven fibers in 4 ERMs (2 dERMs, 2 iERMs) and folded tissue in all the remaining thicker membranes (Figs 1 and 2). All grade II ERMs showed a folded structure at SEM.

Using qualitative SEM/EDS analysis, we identified 15 elements. Of these, aluminium, carbon, and osmium were excluded, because of their presence on the slides. Sulphur was the most frequent element, as it was found in 80% of the samples (9 dERMs and 7 iERMs), followed by sodium (50%, 6 dERMs and 4 iERMs) and phosphorus (45%, 6 dERMs and 3 iERMs) (Figs 1 and 2). The remaining nine elements (silicon, iron, calcium, potassium, magnesium, iodine, manganese, bromine and titanium) were less frequent (Table 1).

**Table 1:** Percentage of frequency of different elements in ERMs.

Element	Frequency of detection
Sulphur (S)	80%
Sodium (Na)	50%
Posphorus (P)	45%
Silicon (Si)	20%
Calcium (Ca)	10%
Iron (I)	10%
Potassium (K)	5%
Magnesium (Mg)	5%
Manganese (Mn)	5%
Iodine (I)	5%
Bromine (Br)	5%
Titanium (Ti)	5%

No statistically significant difference was found between the percentage of sulphur, sodium and phosphorus in diabetic versus idiopathic ERMs (P >0.05).

The number of cases with smooth appearance at SEM was too small to assess any statistically significant difference in the distribution of different elements, when compared with the remaining ERMs. However, we found a trend of sodium to be more frequent in ERMs characterized by folded layers at SEM examination, accounting for the 90% of the ERMs in which the sodium was detected.

## Discussion

To our knowledge, this is the first study analyzing both the mineral composition and anatomical ultrastructure in ERMs using SEM/EDS.

Previous studies examined the cellular layers of ERMs, using tissue cultures and SEM in order to detect the cellular phenotypes involved in the formation of these membranes [20,21]. In particular, recently Azzolini et al. identified different layers of various materials in iERMs [15].

Our study showed a novel finding: the sulphur is the most frequent element in the ERMs. This element is known to be present in significant amounts in proteins, as component of cysteine and methionine [22]. The detection of sulphur may be attributed to the several proteins involved in the pathophysiology of ERMs. First, glial fibrillary acidic protein (GFAP), a specific intermediate filament protein. Indeed, previous studies have demonstrated that glial cells, in particular Müller cells, were the predominant cell type in ERMs [12] and GFAP is the main component of their cytoskeleton [23]. Moreover, GFAP is overexpressed as consequence of damage or stress to the retina, including ERM [24–29]. Second, fibronectin, laminin and vitronectin, that are glycoproteins of the extracellular matrix. These proteins are involved in ERM formation (cellular adhesion, migration, and phenotype differentiation at the vitreoretinal interface) and contain disulfide bonds [30,31]. Third, secreted proteins acidic and rich in cysteine: SPARCs, glycoproteins with adhesive functions located on the basal surface of RPE cells [32,33]. During ERM development, SPARCs reduce the adhesion between RPE and Bruch’s membrane, allowing RPE cells to migrate to the vitreoretinal interface where they de-differentiate into a fibroblast-like cells [34]. At last, metallothioneins, cysteine-rich proteins over-produced by the retina under oxidative stress conditions and involved in ERM formation [7–9].

In this study, the sodium was detected in 50% of samples, mainly in the thicker ERMs (with folded tissue at SEM examination). Recent studies demonstrated that Na^+^ pumps and Na^+^ -dependent ion transporters in astrocytes, microglia and oligodendrocytes regulate Na^+^ homeostasis, modulating glia activity in both physiological conditions and neurological diseases [35–37]. Moreover, Na^+^ signalling increases as consequence of tissue damage [38]. After the ERM formation, in response to chronic insults, such as increased oxidative stress, Na^+^ signalling may contribute to glial activation, cell migration, and gliosis determining the further development of ERMs. This process may explain the higher detection of sodium in thicker ERMs.

Lastly, the phosphorus was found in 45% of ERMs. This finding may be attributed to the activation and proliferation of Muller cells, that need high amount of adenosine 5ʹ triphosphate (ATP) for DNA synthesis [39,40]. The phosphorus is the main component of ATP.

The main limitation of this study is the small sample size.

In conclusion, our study identified traces of several elements in ERMs, a useful and interesting finding to understand their functional role in the physiopathology of this disease and, particularly, in glial activation.
